# Case report: Infective endocarditis caused by *Brevundimonas vesicularis*

**DOI:** 10.1186/1471-2334-6-179

**Published:** 2006-12-29

**Authors:** Mei-Li Yang, Yen-Hsu Chen, Tun-Chieh Chen, Wei-Ru Lin, Chun-Yu Lin, Po-Liang Lu

**Affiliations:** 1 Department of Internal Medicine, Kaohsiung Medical University Hospital, Taiwan; 2 Department of Laboratory Medicine, Kaohsiung Medical University Hospital, Taiwan; 3 Graduate Institute of Medicine, Kaohsiung Medical University, Kaohsiung, Taiwan

## Abstract

**Background:**

There are few reports in the literature of invasive infection caused by *Brevundimonas vesicularis *in patients without immunosuppression or other predisposing factors. The choice of antimicrobial therapy for bacteremia caused by the pathogen requires more case experience to be determined.

**Case presentation:**

The case of a 40-year-old previously healthy man with subacute endocarditis proposed to be contributed from an occult dental abscess is described. The infection was found to be caused by *B. vesicularis *on blood culture results. The patient recovered without sequelae after treatment with ceftriaxone followed by subsequent ciprofloxacin therapy owing to an allergic reaction to ceftriaxone and treatment failure with ampicillin/sulbactam.

**Conclusion:**

To our knowledge, this is the first report of *B. vesicularis *as a cause of infective endocarditis. According to an overview of the literature and our experience, we suggest that third-generation cephalosporins, piperacillin/tazobactam, and ciprofloxacin are effective in treating invasive *B. vesicularis *infections, while the efficacy of ampicillin-sulbactam needs further evaluation.

## Background

*Brevundimonas *(formerly *Pseudomonas*) *vesicularis *is an aerobic nonsporulating and nonfermenting gram-negative bacillus [1] that has been isolated from the external environment [2-5] and human clinical specimens [6-15]. There have been only eight descriptions of infections in human beings caused by the microorganism, with only four cases of bacteremia reported in the literature [Table 1, [8-15]]. Five cases of *B. vesicularis *infection were attributed to community sources [10,12-15] and three to nosocomial sources [8,9,11]. Such underlying diseases as autoimmune disorders associated with long-term steroid use, end-stage renal disease treated by hemodialysis, and sickle cell anemia with functional asplenia contributed to *B. vesicularis *infections in 6 of the 8 reported cases due to the patients' immunocompromised state [Table 1, [8-12,15]]. *B. vesicularis *infection has therefore been classified as opportunistic [8-12,15]. We report here a case of infective endocarditis caused by *B. vesicularis *in a patient without comorbid disease. The low number of cases reported of *B. vesicularis *infection in humans limits the body of knowledge on the spectrum of disease caused by this pathogen as well as optimal treatment regimens. The treatment course of the current case and previous cases of *B. vesicularis *infection is further discussed.

**Table 1:** Summary of reported cases of *Brevundimonas vesicularis *infection in humans

**Reference**	**Age (y)/sex**	**Underlying disease**	**Clinical presentation**	**Treatment/duration**	**Outcome**
(8)/NI	54/F	SLE, autoimmune hepatitis, steroid therapy	Necrotizing cellulitis	Tobramycin + ceftazidime/30d	Survival
(9)/NI	42/F	Mitral valve stenosis s/p mitral valve replacement	Septicaemia	Piperacillin - tazobactam/14d	Survival
(10)/CA	5/M	Sickle cell anemia	Pneumonia	Ceftriaxone + gentamicin/10d	Survival
(11)/NI	59–67/both	Hemodialysis	Septicaemia	Cefotaxime + tobramycin/unknown	Survival
(12)/CA	60/M	Trauma	Botryomycosis	Cefuroxime- axetil/4 months	Survival
(13)/CA	38/M	No	Acute tonsillitis	amoxicillin- clavulanate/3d	Survival
(14)/CA	1.25/F	No	Septic arthritis	cefuroxime/12d + oral cephalexin/14d	Survival
(15)/CA	55/M	Continuous ambulatory peritoneal dialysis	peritonitis	ceftazidime/3d, ceftazidime + vancomycin/4d, ciprofloxacin + aztreonam/14d	Survival
current case/CA	40/M	No	Subacute endocarditis	cefazolin + gentamicin/3 d, Ampicillin/sulbactam + gentamicin/5 d, Ceftriaxone + gentamicin/7 d, ciprofloxacin/30 d	Survival

Abbreviations:

CA = community-acquired infection;

NI: nosocomial infection. Nosocomial infections are those that originate or occur in a hospital if they first appear 48 hours or more after admission or hospital-like settings.

## Case presentation

**Figure 1:**
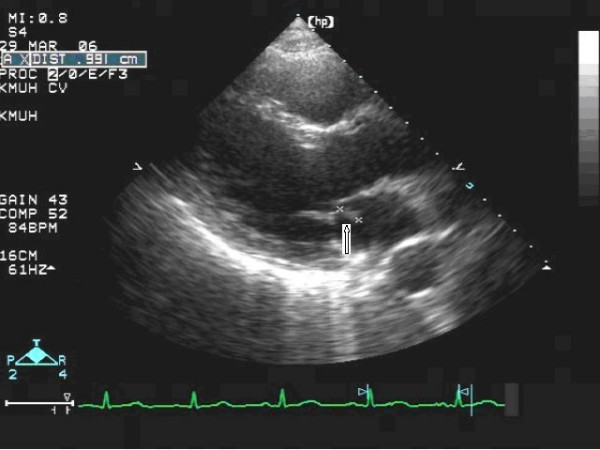
Transthoracic echocardiogram showing vegetation (0.9 cm) over the anterior leaflet of mitral valve.

**Figure 2:**
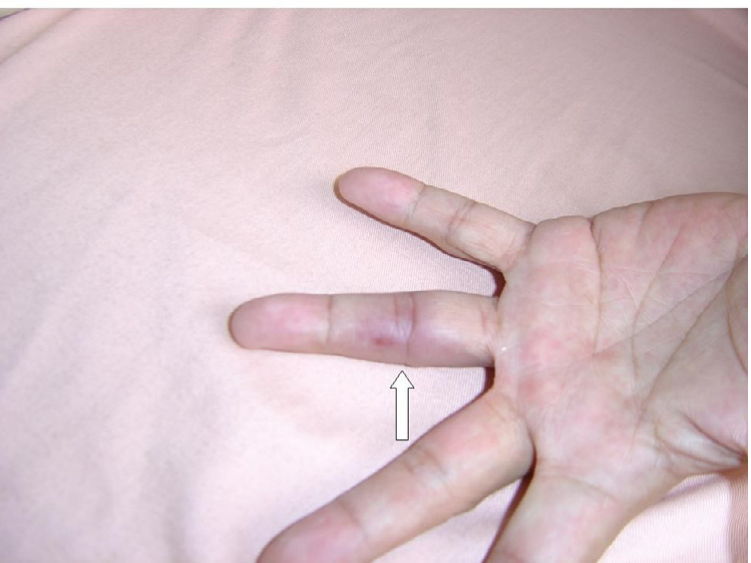
Osler's node on the fourth digit of the left hand.

**Figure 3:**
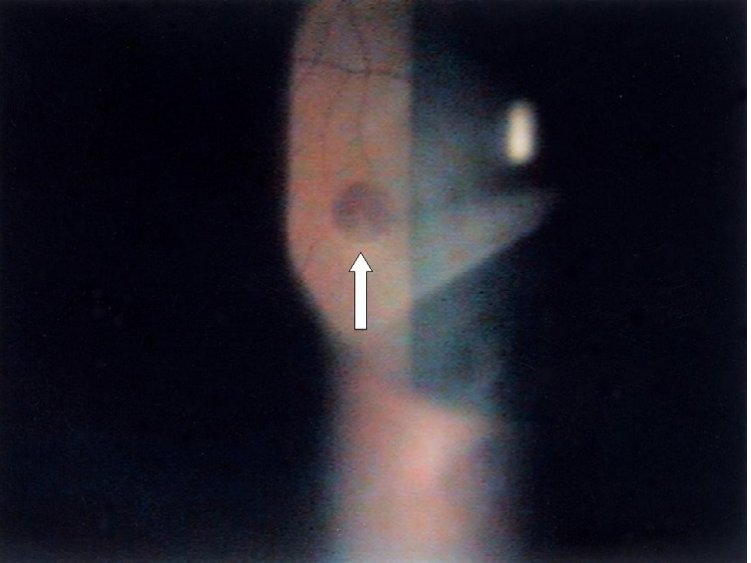
Roth's spot on the retina of the left eye.

The patient was a 40-year-old male admitted for chronic cough and a 2-month history of intermittent afternoon fever. He denied both a history of traveling and contact with animals over the previous 3 months. Associated symptoms included general malaise, anorexia, and the loss of 6 kg body weight within the previous 2 months. On admission, the vital signs were shown as blood pressure: 124/68 mmHg; pulse rate: 80/min; respiratory rate: 22/min; body temperature: 38.5°C. Physical examination revealed a pansystolic murmur over the apex and left lower sternal border. An abscess in a molar cavity that required debridement was found, although the patient had not complained of dental pain. Laboratory tests revealed a WBC count of 9250/μL with elevated neutrophils (82.7%); normocytic anemia with a hemoglobin level of 10.4 g/dL; an elevated ESR level (123 mm/1 hr); and abnormal liver function with AST/ALT of 49/79 IU/L. A chest x-ray film revealed increased infiltration over the right upper and lower lobes, and abdominal sonography showed mild splenomegaly. Diagnosis of pulmonary tuberculosis was excluded by negative results from serial acid-fast stain and culture of *Mycobacterium tuberculosis*. Transthoracic echocardiography revealed a vegetation with size of 0.9 cm over the anterior leaflet of mitral valve, and moderate mitral regurgitation. However, the cardiac systolic and diastolic functions were normal with the ejection fraction of 78.9%, fraction shortening of 47.8%. Ultimately, definite subacute endocarditis was diagnosed according to the Duke criteria, including two blood culture results of *B. vesicularis*; the presence of vegetation on the mitral valve revealed by an echocardiogram (Fig. 1); and such clinical and laboratory features as fever, splenomegaly, heart murmur, anemia, Osler's node (Fig. 2),,and Roth's spot (Fig. 3).

The pathogen was identified as *B. vesicularis *on the basis of the biochemical characteristics tested by the API ID 32 GN automatic identification system (bioMérieux, Marcy I' Étoile, France). These included the characteristics of positive activity of D-maltose/D-glucose oxidation; negative activity of inositol/D-sorbitol/L-arabinose oxidation; negative activity of L-alanine/capric acid/potassium 5-ketogluconate/trisodium citrate decarboxylation; negative activity of nitrate reduction and indole production. The organism was susceptible to amikacin, gentamicin, piperacillin, aztreonam, cefepime, meropenem, netilmicin, ampicillin, amoxicillin, ciprofloxacin, cefazolin, cefmetazole, ceftazidime, cefotaxime, ceftriaxone, and ticarcillin, according to the susceptibility test performed by the disc diffusion method with interpretation criteria for *Pseudomonas *[16]. The patient was treated with a combination of cefazolin (2 gm/8 hr) and gentamicin (80 mg/8 hr) as empiric antibiotics during the first 3 days and fever subsided gradually.

Ampicillin/sulbactam (3 gm/6 hr) was used alternatively for covering the infection of oral anaerobes while the patient underwent removal of the abscessed tooth. Treatment with amipicillin/sulbactam failed, however; the patient developed a flare-up of fever, the appearance of a new Osler's node on the fourth digit of the left hand (Fig. 2), and pain in the right femur with right inguinal lymphadenopathy. Treatment with a combination of ceftriaxone (2 gm/12 hr) and gentamicin (80 mg/8 hr) was administered subsequently. The femoral-inguinal symptoms and fever improved gradually with the antimicrobial therapy and therefore were ascribed to embolism. Although the clinical symptoms and signs improved after use of these antibiotics, ciprofloxacin (400 mg/12 hr) was administered as an alternative due to the eruption of a skin rash from an allergic reaction after 7 days' treatment with ceftriaxone. Ultimately, the patient received the treatment regimen for one month and was discharged. There were no sequelae at 6-month follow-up and the mitral vegetation disappeared in the follow-up echocardiogram performed one month after discharge.

## Conclusion

Subacute infective endocarditis commonly involves heart valves already damaged in some way and is most often caused by one of the *Viridans streptococci*, such as *Streptococcus sanguis*, *S. mutans*, *S. mitis*, or *S. milleri*, which normally live in the mouth and throat. To the best of our knowledge, this is the first presentation of *B. vesicularis *manifesting as subacute endocarditis of the native valve. The dental cavity, which was the only infective focus noted, was proposed to be the origin of the infection even though no direct evidence of *Brevundimonas *was isolated from the dental abscess. *B. vesicularis *has been isolated from pus culture with tonsil swabs [13], suggesting the existence of the pathogen in the oral cavity. Furthermore, in the cases of tonsillitis and septic arthritis, the subjects had been immunocompetent before the occurrence of *Brevundimonas *infection [13,14]. Therefore, the clinical significance of the pathogen should not be confined solely to infections in immunocompromised hosts.

According to researchers at the CDC, *B. vesicularis *is highly sensitive to all aminoglycosides (98%), piperacillin (100%), carbenicillin (94%), and ceftizoxime (94%), with moderate susceptibility to cefamandole (50%) and cefoxitin (75%), and lower susceptibility to penicillin (12%) and ampicillin (22%) [17]. The antibiograms of isolates from clinical cases were variable, however; most were determined by the disc diffusion method [[8,9,13], Table 2]. Our reported case was treated with cefazolin and gentamicin as empiric antibiotics initially, followed by amipicllin/sulbactam, then ceftriaxone and gentamicin, followed with ciprofloxacin. The failed clinical response in our case to ampicillin/sulbactam therapy even though susceptibility testing has demonstrated that the organism is susceptible to ampicillin/sulbactam, indicates that results from the disc diffusion method with reference to *Pseudomonas *species are not suitable in guiding antimicrobial therapy for *B. vesicularis *endocarditis. For endocarditis, minimum inhibitory concentration (MIC), minimum bactericidal concentration and serum bactericidal titer determination are appropriate to guide antimicrobial therapy. Our report has the limitation of lacking MIC determination as the antimicrobial susceptibility testing method. It is difficult to decide on the best antimicrobial for invasive infection due to *B. vesicularis *because of the low number of reported cases in humans caused by this pathogen; the fact that isolates come from various countries or findings from different laboratories; and the lack of standarized testing. Five of the reported cases [[8,10-12,14], Table 1] were successfully treated with a second- or third-generation cephalosporin with or without an aminoglycoside; two cases were treated with a β-lactam/β-lactamase inhibitor [[9,13], Table 1]; Two cases were treated with ciprofloxacin alone or combined with a monobactam [current case, [15], Table 1]. Our experience in this case and previous cases of bacteremia suggest that a third-generation cephalosporin, piperacillin/tazobactam, aztreonam and ciprofloxacin are effective in treating infection due to *B. vesicularis*. Though subacute bacterial endocarditis caused by *Pseudomonas *spp. should be appropriately treated for 6 weeks [18], our reported case was successfully treated with four-week antimicrobial therapy. To decide the optimal duration of therapy for *B. vesicularis *endocarditis, more experience with such case is required.

**Table 2:** In-vitro antibiotic susceptibility of *Brevundimonas vesicularis*

**Reference**	Current Case*	**Choi**^# ^**(15)**	**Sofer**^# ^**(14)**	Chi* (13)	Gilad* (9)	**Oberhelman‡ (10)**	Planes* (8)
**Year**	**2006**	**2006**	**2006**	**2004**	**2000**	**1994**	**1992**
**Antibiotics**							
Ampicillin	S	N	S	R	R	N	S
Piperacillin	S	S	S	N	S	S	R
Mezlocillin	N	N	N	N	S	S	N
Carbenicillin	N	N	N	N	N	S	N
Amoxicillin/clavulanate	S	N	N	N	S	N	N
Piperacillin/tazobactam	S	N	N	S	S	N	N
Cefazolin	S	N	S	R	N	N	S
Cefuroxime	N	N	S	S	R	N	N
Cefoxitin	N	N	S	N	N	N	S
Cefotaxime	S	N	S	S	N	N	S
Ceftriaxone	S	N	S	S	R	S	S
Ceftazidime	S	S	S	R	R	S	R
Gentamicin	S	S	S	R	S	S	S
Tobramycin	N	N	S	R	S	N	S
Amikacin	S	N	S	S	S	N	S
Doxycycline	N	N	S	S	N	N	S
Chloramphenicol	N	N	N	S	N	N	S
Cotrimoxazole	S	N	S	S	S	N	S
Imipenem	N	S	N	S	S	N	N
Aztreonam	S	S	N	R	R	N	N
Ciprofloxacin	S	S	S	R	R	S	N

Abbreviations: S = susceptible; R = resistant; N = not determined

* Susceptibility was determined by disc diffusion.

‡ Susceptibility was determined by automatic microdiffusion.

# Susceptibility was determined by an unknown method.

In conclusion, since cases of infection due to *B. vesicularis *are reported with increasing frequency, our endocarditis case report reinforces the hypothesis that bacteria can cause invasive disease in a person without predisposing disease. More treatment experience and more exact results from antimicrobial susceptibility testing are required to improve on present treatment regimens for invasive *B. vesicularis *infection.

## List of abbreviations

ALT: alanine aminotransferase

AST: aspartate aminotransferase

CDC: Centers for Disease Control and Prevention, Atlanta, GA, U.S.A.

ESR: erythrocyte sedimentation rate

IU/L: international units per liter

SLE: systemic lupus erythematosus

WBC: white blood cell

MIC: minimum inhibitory concentration

## Competing interests

The author(s) declare that they have no competing interests.

## Authors' contributions

ML Yang and PL Lu took care the patient during hospitalization and prepared the manuscript. YH Chen, TC Chen, WR Lin and CY Lin participated in the the diagnosis and treatment of the disease. All authors read and approved the final version of the manuscript.

## Pre-publication history

The pre-publication history for this paper can be accessed here:


